# The Role of Pro-Resolving Lipid Mediators in Colorectal Cancer-Associated Inflammation: Implications for Therapeutic Strategies

**DOI:** 10.3390/cancers12082060

**Published:** 2020-07-26

**Authors:** Federica Ungaro, Silvia D’Alessio, Silvio Danese

**Affiliations:** 1 IBD Center, Laboratory of Gastrointestinal Immunopathology, Humanitas Clinical and Research Center, Rozzano, 20089 Milan, Italy; silvia.dalessio@hunimed.eu (S.D.); sdanese@hotmail.com (S.D.); 2 Department of Biomedical Sciences, Humanitas University, Rozzano, 20089 Milan, Italy

**Keywords:** colorectal cancer, cancer-associated inflammation, lipids, resolution of inflammation, PUFAs

## Abstract

Inflammation is a recognized hallmark of cancer that contributes to the development and progression of colorectal cancer (CRC). Anti-inflammatory drugs currently used for the treatment of CRC show many adverse side effects that prompted researchers to propose the polyunsaturated fatty acids-derived specialized pro-resolving mediators (SPMs) as promoters of resolution of cancer-associated inflammation. SPMs were found to inhibit the CRC-associated pro-inflammatory milieu via specific G-coupled protein receptors, although clinical data are still lacking. This review aims to summarize the state-of-the-art in this field, ultimately providing insights for the development of innovative anti-CRC therapies that promote the endogenous lipid-mediated resolution of CRC-associated inflammation.

## 1. Introduction

Colorectal cancer (CRC) incidence and mortality rates consistently vary throughout the globe, since its onset depends on different factors, such as environment, dietary, and daily habits, as well as genetics [1]. The incidence of CRC is higher in industrialized and economically advanced countries, where the western diet is prevalent [1]. According to the International Agency for Research on cancer, globally, CRC is the third most commonly diagnosed cancer in males and the second in females, being also the fourth cause of death worldwide, with 1.8 million new diagnoses and about 800,000 deaths in the last years. Although the mortality rate is still high, CRC-related death has progressively been declining during the last three decades due to prevention, early diagnosis, and effective treatments [2]. 

The etiopathogenesis of CRC consists of four key stages [1], comprising firstly of initiation when an irreparable DNA damage in bowel wall epithelial cells triggers neoplastic transformation. Then, during the *promotion stage*, cells start to aberrantly proliferate, leading to the neoplasm. Such an uncontrolled cell proliferation favors the initiation of other genetic and epigenetic alterations that can transform cells from benign to malignant cancerous cells and can endow them with invasive and metastatic potential. This is the progression stage. The last phase, the metastasization process, is characterized by cancer cell detachment from the bowel wall and their diffusion throughout the blood and lymphatic circulations, finally reaching distant organs, such as the liver, where malignant cancerous cells adapt and colonize the hepatic parenchyma. The liver is the most common site of metastasization in CRC patients because of portal circulation functioning as a direct flow between the liver and intestine [3]. However, CRC can metastasize, to a lesser extent, to the lungs and bones depending on the primary cancerous lesions, whether in the colon or the rectum. In this regard, metastatic distribution analysis of data extrapolated from the Surveillance, Epidemiology, and End Results Program (SEER) database revealed that, while colon cancer-derived metastasis preferentially reached the liver, rectum cancer had a higher incidence of lung and bone metastasis. Moreover, CRC patients with lung metastasis showed an increased risk of metastasization to bones or the brain by comparison with patients without evidence of lung metastasization [4].

Although the majority of CRC cases are sporadic (accounting for 60 to 65% of all diagnoses), CRC has also hereditary components (about 35–40%) [1]. The genetic risk of CRC is associated with rare but high-penetrance germline mutations in susceptibility genes, such as *MLH1* and *APC*, that cause hereditary nonpolyposis colorectal cancer (HNPCC, also known as Lynch syndrome) and familial adenomatous polyposis (FAP), respectively [5]. However, genetic mutations lead to colorectal carcinogenesis in 9.9% of all diagnosed CRC [5]. Additionally, less than 1% of CRC hereditability might be explained by low-penetrance genetic variations that increase CRC risk, even if such predispositions are fostered by environmental factors [1]. 

With CRC risk increased and accelerated by external factors, such as the environment, daily habits (i.e., smoking), and diets (such as high red-meat consumption), the route to prevention includes several suggestions to lower the probability of CRC occurrence. For example, recommendations for decreased CRC risk are smoking cessation, healthy diet, and regular exercise that can prevent the development of CRC [6]. Also, the regular use of vitamin supplements and hormone replacement therapy have been associated with reduced risk for CRC [6].

Of note, after treatment of CRC, several factors have been associated with improved outcomes and decreased risk of colorectal cancer-related death. As a piece of evidence, patients with a healthy lifestyle, such as smoking cessation; daily physical activity of at least 30 min; consumption of milk, whole grains, fresh fruits, tree nuts, and vegetables; and intake of calcium and fibers had a high rate of survival against advanced CRC [7], indicating that nutrients and good quality of life may promote the resolution of CRC.

Another risk factor for CRC onset is inflammation, so patients with Inflammatory Bowel Disease (IBD) display an increased propensity to colorectal carcinogenesis up to 2.4 fold compared to the general population [8]. Such a predisposition is due to the pro-inflammatory milieu existing in IBD patients’ gut. In this regard, it is well established that chronic inflammatory conditions predispose normal cells to progressing from indefinite dysplasia to cancer, passing through low-to-high grade dysplasia. However, due to the low incidence of IBD and the use of anti-inflammatory treatments along with prophylactic colectomy in IBD patients, this carcinogenetic pathway explains less than 2% of all CRC [1,9]. 

Whatever the cause, all CRC cases show complex pathogenesis, where inflammation represents one of the most important hallmarks [9] so that targeting tumor-associated inflammation is a strategy for colon cancer prevention [9]. Consistently, anti-inflammatory treatments have been used for prophylaxis and have shown efficacy in decreasing cancer morbidity [10]. Most of the anti-inflammatory agents used are nonsteroidal anti-inflammatory drugs (NSAIDs), which inhibit Cyclooxigenase (COX) enzymes, are able to metabolize arachidonic acid (AA), and produce AA derivatives, such as prostaglandins (PGs) [11]. Among them, aspirin showed efficacy in chemoprevention of CRC, reducing the risk up to 50% [12,13]. Naproxen, another NSAID, has shown efficacy in anticancerous treatment by inhibiting the production of PGE2 in CRC [14]. Another example of an NSAID used in CRC-derived inflammation is sulindac, which has been shown to reduce CRC inflammation via the inhibition of COX-1/COX-2-dependent and -independent effects [14]. Moreover, sulindac combined with atorvastatin has even been proven to inhibit tumor growth [15].

Other anti-inflammatory drugs are celecoxib, inhibiting COX-2, and licofelone, which significantly decreased COX and 5-LOX activities. Both celecoxib and licofelone showed chemopreventive potentials against colon cancer [14]. These clinical outcomes suggested that anti-inflammatory treatment contributes to the resolution of CRC-associated inflammation by dampening the pro-inflammatory milieu and by triggering pro-resolving pathways [16]. By contrast, despite their beneficial role in chemoprevention in CRC, NSAIDs cause many side effects [17]. For example, naproxen, sulindac, and aspirin have been shown to induce gastroduodenal damage, hemorrhage, enteropathy, and ulceration. Moreover, aspirin as well as celecoxib provoke cardiovascular side effects [14]. Therefore, the discovery of alternative therapies that can avoid the anti-inflammatory drug-induced side effects would be of extreme importance.

All events in cancer-associated inflammation are orchestrated by a plethora of innate-immunity players (neutrophils, macrophages, innate lymphoid cells, intraepithelial lymphocytes, myeloid-derived suppressor cells, and natural killer cells); adaptive immune cells (B and T lymphocytes); intestinal epithelial cells (i.e., Paneth cells); and other cells part of the tumor microenvironment such as carcinoma-associated fibroblasts (CAFs), vascular endothelial cells/pericytes, and mesenchymal cells [18].

Of course, as for inflammatory conditions, these cells may interact and “talk” with each other through a set of cytokines, chemokines, and other growth factors triggering specific receptors. Based on the pathway activated, these complex networks may display anti- or pro-tumorigenic functions [9]. 

In the last decade, inflammation has been discovered as a distinct event from its resolution. If in the past the resolution of inflammation was considered a passive process, where pro-inflammatory signals just dilute and dissipate over time, recently, pro-resolving pathways have been shown to actively, spatially, and timely regulate the pro-inflammatory phase of inflammation [19,20]. In this context, specialized pro-resolving lipid mediators (SPMs) derived from polyunsaturated fatty acids (PUFAs), including ω-3 and ω-6 long-chain fatty acids, were demonstrated to be in charge of resolving inflammation and in chronic inflammatory conditions [19].

Because of the tight relationship between tumor and inflammation, SPMs have been recently proposed as potent bioactive molecules with antitumor activity in various organs [21,22,23]. Moreover, SPM analogs inhibited Vascular Endothelial Growth Factor (VEGF)-induced endothelial permeability by stabilizing the Vascular Endothelial (VE)-cadherin/ß-catenin-dependent adherens junctions to protect patients from tumor extravasation across endothelial barriers [24].

Since the resolution code, made of lipid molecules and their enzymatic route of production, has been extensively reviewed elsewhere [19,25], here, we will discuss recent findings on the role of pro-resolving lipid mediators in CRC-associated inflammation, pointing out the lipid-mediated resolution of inflammation as a possible and promising therapeutic target for CRC therapy. 

## 2. Literature Search Strategy

A large and comprehensive literature through Medline (Pubmed) and Google has been conducted to identify all relevant citations published within the last thirty years by using the following terms either alone or in combination: “colon”, “carcinoma”, “colon carcinoma”, “colorectal cancer”, “colorectal cancer inflammation”, “resolution of inflammation colorectal cancer”, “SPM colorectal cancer”, “omega-3 colorectal cancer”, “colitis-associated cancer”, and “PUFA”. Highly regarded relevant articles were not excluded a priori. Only studies exploring cellular, molecular, and clinical characteristics of colorectal cancer, inflammation, and its resolution have been selected. We also searched the reference lists of key review articles for additional papers we considered to be relevant to this manuscript.

## 3. The CRC-Associated Pro-Inflammatory Milieu

**Figure 1:**
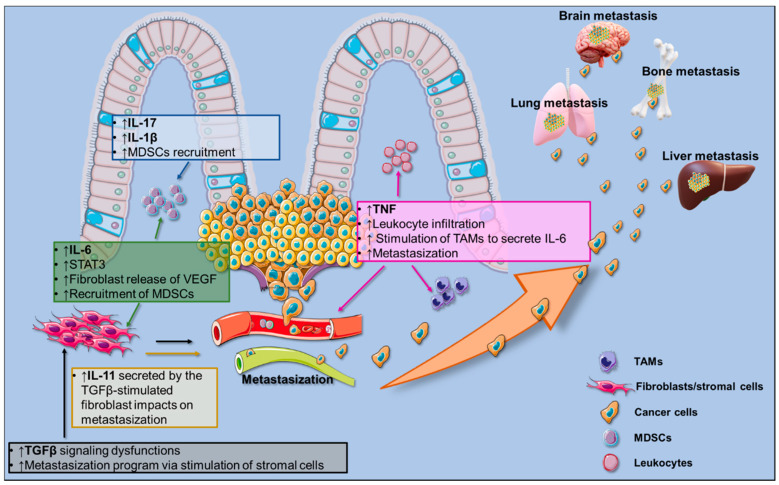
Pro-inflammatory milieu orchestrating colorectal cancer (CRC) growth and metastasization to the liver, lungs, and bones. Text boxes list specific cytokine (Interleukin (IL)-17, IL-1β, Tumor Necrosis Factor Alpha (TNFα), IL-6, and IL-11)-associated functions; arrows indicate cellular targets of CRC-associated cytokines. The illustration was made with SMART Servier Medical Art free images (https://smart.servier.com/).

Human studies showed that pro-inflammatory components, consisting of both cells and molecules, are present also in the tumor microenvironment, suggesting that tumor-associated inflammation may drive cancer development and progression in the gut [26] (Figure 1). This evidence was further corroborated by animal models of CRC, employing either Azoxymethane (AOM)/Dextran Sodium Sulfate (DSS) treatment or the genetic modification based on *Apc* gene ablation/mutation. Here, we will list a series of the main pro-inflammatory components that characterize the CRC pathogenesis.

### 3.1. Cellular Components of the CRC Microenvironment 

Among the cellular components, both immune and nonimmune cells infiltrate CRC tissues [27] and actively participate in tumor-promoting inflammatory pathways. During CRC pathogenesis, epithelial cells are recognized as the master players in driving the carcinogenetic process, since the transformed epithelium not only contributes to the neoplastic lesion growth but also fails to act as a barrier against microbiome species that then become essential for the induction and maintenance of tumor-promoting inflammation [28]. Also, the secretory Paneth cells, located in the intestinal crypt base, were proposed as participants to intestinal inflammation [29] and their presence in intestinal adenomas was correlated with increased risk of colorectal neoplasia [30], indicating that they can play in concert with the pro-inflammatory milieu to fasten the CRC initiation and progression [9].

The immunophenotypic signature infiltrating the tumor areas is multifaceted. This is made of innate immune cells, such as macrophages and dendritic cells, all involved in the antitumor response [31]. These cells are designated for the immune surveillance and the recruitment of cells of the adaptive immune response. In the tumor microenvironment, neutrophils deserve much more attention, since they participate in the inflammatory process and most notably are also pivotal for the resolution of inflammation [32]. They are physiologically deputed to produce and release pro-inflammatory mediators, such as the leukotriene B4 (LTB4) derived from the AA during the acute phase of the inflammatory response, but they switch to an anti-inflammatory state releasing SPMs (i.e., lipoxins, resolvins, and protectins) to induce the resolution of inflammation [33,34]. Also, neutrophils undergoing apoptosis during acute inflammation can stimulate macrophages into a pro-resolution phenotype, reducing the inappropriate inflammatory response further [35]. 

When an inflammatory insult persists, mechanisms of immune surveillance may fail, with the concurrent recruitment of immunoregulatory cells [36], including myeloid-derived suppressor cells (MDSCs), T regulatory (Treg) cells, type 2 macrophages, and other cancer-associated cell types [37], that altogether sustain tumor cell growth [27]. Additionally, in intestinal mucosa with prolonged inflammation, neutrophils continuously accumulate. Apoptotic neutrophils, not cleared out by macrophages, undergo secondary necrosis and release the contents of intracellular granules, which can induce pathological tissue damage [35]. 

Last but not least, in this complex scenario, B lymphocytes, plasma cells, eosinophils, and mast cells cooperate with both pro-inflammatory and immunosuppressing cells during CRC pathogenesis [38].

Angiogenesis is one of the most impressive hallmarks of CRC [39,40,41,42] because, at the basis of tumor growth and metastasization, the latter occurring mainly via lymphatics, CRC angiogenesis is driven by VEGF signaling acting through tyrosine receptor kinases, VEGFR1 and VEGFR2 are implicated in blood vessel development and branching, and VEGFR3 is implicated in lymphangiogenesis [43], so that antiangiogenic therapies resulted effective in CRC treatment, mainly in patients with liver metastasis [43].

It is noteworthy that the tumor microenvironment is characterized not only by immune cells and vasculature but also by CAFs and mesenchymal stem cells (MSCs), the latter observed to migrate to the tumor site, to transform into CAFs [44], and to influence tumor development and progression in CRC by expressing VEGF [45,46].

Of note, CAFs cooperate with other cells to constitute a CRC pro-inflammatory microenvironment by secreting the pro-inflammatory cytokines and by stimulating angiogenesis [47]. 

Based on the evidence listed above, it is clear that the tumor-associated inflammation is a concert of cells directed and coordinated by specific molecules and signals secreted within the tumor microenvironment and is capable of driving carcinogenic processes. In the next subsection, we will highlight the major actors involved in such a complex scenario. 

### 3.2. Pro-Inflammatory Signals in CRC

One of the most studied signal molecules involved in expansion, invasion, and metastasization of tumor cells is Tumor Necrosis Factor-alpha (TNFα). TNFα belongs to a large family of cytokines playing roles in a plethora of processes [48], is commonly considered a tumor promoter, and is found to orchestrate both initiation and metastasization processes in animal models of CRC [49,50], also independently of inflammation [51,52]. Its role in tumor growth and progression was also evidenced in human samples, where TNFα levels were directly correlated with CRC progression [53]. However, some discrepant results showing TNF superfamily cytokines to be protective against CRC development might be explained by the use of different animal and cellular models that probably neglect the concomitant contribution of other signaling routes to the tumorigenic process [54,55]. 

Although not a proper pro-inflammatory signal, Transforming growth factor β (TGFβ) deserves attention in this context since it is a multifunctional cytokine that, on one hand, can induce apoptosis and differentiation of intestinal epithelial cells as well as wound healing and, on the other hand, has a role in cancer [56], acting through the binding to its receptors TGFβR1 and TGFβR2. Its relationship with CRC relies on the discovery of the high rate of CRC-associated mutations in genes encoding for the TGFβ signaling-related proteins [57] that lead to molecular dysfunctions, rendering the TGFβ a growth stimulator, causing cancer progression and malignant transformation [58] by programming the metastasization process in locally advanced CRC [59,60]. In line with this, TGFβ can also predict the risk of relapse in patients with CRC [60]. 

One of the master regulators of CRC-associated inflammation is Interleukin 1 β (IL1β). IL1β participates in the activation of Nuclear Factor Kappa-light-chain-enhancer of activated B cells (NF-κB)-driver molecular machinery [61] and, for this reason, was classified as pivotal signal molecule contributing to CRC pathogenesis in both preclinical studies, exploiting either the AOM/DSS [62] or the genetic models of CRC [52] and human samples [63], where polymorphisms in its gene and receptors are associated with this disease [64].

Together with IL1β, Interleukin 6 (IL6) represents the prototype of inflammatory cytokines and exhibits a plethora of roles, ranging from regulation of pro-inflammatory signaling, proliferation, and angiogenesis to modulation of tumorigenesis and metastasis [65,66]. In this regard, IL6 has been identified as a key player in CRC [67] and colitis-associated cancer (CAC) [65], with evidence further supported by animal models using either the AOM/DSS-induced or the genetic models of colorectal carcinogenesis [68,69,70]. Furthermore, tumor-associated macrophages (TAMs) participate in CRC metastasization through the TNF-α mediated secretion of IL6 [71,72], which was also found to directly promote the accumulation of MDSCs in tumors [73]. Notably, among the IL6 family members, also interleukin 11 (IL11), produced by TGF-β-stimulated CAFs, was found to play a role in tumor growth and distal metastasization in vivo [60,74].

Last but not least, interleukin 17 (IL17) was shown highly expressed in human samples and was capable of recruiting MDSCs, also increasing their immunosuppressive functions [75,76]. Animal studies strengthened its role in colorectal carcinogenesis. Indeed, Il-17 was found to contribute to tumor formation and growth in vivo*,* also independently of inflammation [77,78,79]. 

The pro-inflammatory milieu depicted so far and evidencing only the most important but not the sole players in CRC-associated inflammation highlights how its blockage can be challenging. In fact, the inhibition of one or a few components of the inflammatory network could subvert the physiology of other districts of the organism not directly involved in the CRC pathogenesis, interfering with the normal functioning of the body and causing side effects. Therefore, to investigate the pathways of the resolution of inflammation, as we are going to discuss shortly, may be successful for the development of innovative therapies able to counteract CRC.

## 4. Pro-Resolving Lipid Mediators: A Brief Overview

As introduced earlier, in the last decades, there has been a significant revolution in the classical paradigm underlying inflammation and its resolution. While in the past the resolving process was considered just a passive and spontaneous consequence of the acute inflammatory response, in recent years, a growing and convincing body of evidence opened new frontiers for the management of inflammation not only in the physiological series of events, like those occurring after infections, but also in chronic inflammatory conditions [80]. In fact, after the discovery of novel and potent endogenous signals activated by the SPMs that spatially and temporally orchestrate the resolving process, the field of therapy for chronic inflammation found a novel possible line of interventions [25]. 

SPMs encompass both ω-3 α-linolenic (ALA) and ω-6 linoleic (LA)-derived bioactive molecules formed in vivo via the cyclooxygenase (COX), lipoxygenase (LOX), and cytochrome P450 (CYP450) monooxygenase pathways [19]. The essential fatty acid ALA, not synthesized by the physiological metabolism and thus been taken up through diet, is converted to eicosapentaenoic acid (EPA), docosapentaenoic (DPA), and docosahexaenoic acid (DHA).

**Figure 2:**
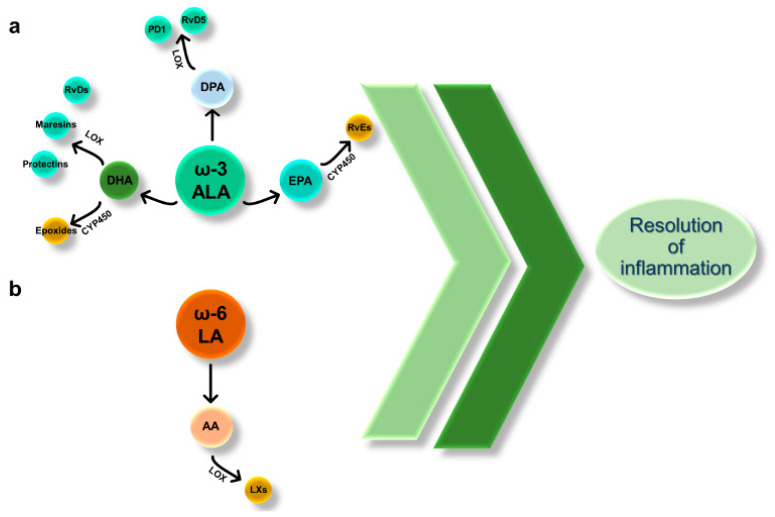
Ω-3 and ω-6-derived lipids enhance the resolution of inflammation. (**a**) Essential fatty acid α-linolenic acid (ALA) is converted to eicosapentaenoic acid (EPA), docosahexaenoic acid (DHA), and Docosapentaenoic acid (DPA). EPA and DHA may be the substrates of CYP450, resulting in the production of E-series resolvins (Rv) and epoxides, respectively. Besides, DHA and DPA are metabolized via lipoxygenase (LOX) to D-series Rvs, maresins, and protectins. (**b**) Essential fatty acid linoleic acid (LA), classified as a ω-6 polyunsaturated fatty acid, can be converted into arachidonic acid (AA). Via the lipoxygenase (LOX) pathway, AA is converted to lipoxins (LXs).

EPA and DHA may be the substrates of CYP450, resulting in the production of SPMs belonging to the class of E-series resolvins (RvE) and epoxides, respectively. Besides, DHA and DPA are metabolized via LOX to other SPMs, including D-series Rv (RvD), maresins, and protectins (Figure 2a). All these EPA-, DHA-, and DPA-derived SPMs have been recognized to harbor pro-resolving properties in inflammatory disorders [19].

LA, like ALA, is an essential ω-6 PUFA, that humans can metabolize to AA. Further, AA can be converted to lipoxins via the LOX pathway (Figure 2b). Lipoxins (LXs) belong to the class of SPMs as those derived from DHA, EPA, and DPA, and their role in counterbalancing inflammation has been extensively reviewed elsewhere [81].

It is noteworthy that, except for LXs, the other ω-6 PUFA-derived metabolites, such as prostaglandins, thromboxanes, and leukotrienes, are conventionally involved in the initiation of inflammatory responses as well as in cancer [82]. On the contrary, most of the ω-3 PUFA-derived molecules seem to promote the resolution of inflammation [83]. 

The general mechanism of action of the SPMs has been largely described in outstanding reviews from Serhan and colleagues, who conducted the pioneering studies in this field [81]. Regarding the role and the action of these lipid mediators in the intestine, several studies highlighted SPM-specific functions (extensively reviewed in Ungaro et al. 2017 [19]). As an example, LXs and RvE_1_ were found to upregulate the bactericidal permeability-increasing protein (BPI) [84] necessary to protect the gut from microbiota-triggered inflammation. Similarly, RvE_1_ triggered the expression of the intestinal alkaline phosphatase [85], important for bacterial homeostasis in the gut. Moreover, both LXs and RvE1 were displayed capable of reducing neutrophil infiltration at the site of inflammation [19]. Also, LXs were displayed to exert antiapoptotic functions on epithelial cells during gut inflammation [86] and treatment of macrophages with LXA4 and its analogs induced a strong enhancement in phagocytosis of apoptotic neutrophils [87]. Finally, concerning maresins (MaR), MaR1 may cause the M1-to-M2 macrophage switch and the direct blockade of neutrophil transmigration and reactive oxygen species production during intestinal inflammation in mice [88]. Overall, these pieces of evidence suggest the strong ability of these lipid molecules to dampen inflammation in the gut. Herein, because of the cooccurrence of either intrinsic (cancer cells that trigger inflammation) or extrinsic (chronic inflammation that promotes cancer) processes promoting cancer development [89], it was reasonable to think that cancer, including CRC, might find new frontiers in therapies exploiting the pro-resolving ω-3 and ω-6 PUFAs and their derivatives to enhance the resolution of tumor-associated inflammation.

## 5. The Resolution of CRC-Associated Inflammation through the ω-3 PUFAs: A Lesson from Clinical and Animal Studies

Cancer, including CRC, is considered a “western country” disease [90] since its occurrence was associated with western dietary habits, characterized by an increased uptake of short-chain fatty acids (SFAs) and trans fatty acids (FAs). This may cause an alteration in essential FA intake and an increase of the ω-6/ω-3 ratio, normally associated with pro-inflammatory conditions [20]. Compelling evidence comes from epidemiological studies that gave prominence to the protective role of ω-3 PUFA against the risk of developing cancer [90], including CRC. In fact, a population-based prospective study revealed the indirect correlation between the dietary intake of marine ω-3 PUFAs and the risk of CRC [91]. Additionally, other clinical studies further confirmed the inverse correlation between CRC risk and ω-3 dietary intake [92,93,94]. Consistently, a high ratio of ω-6 AA/ω-3 EPA-derived epoxyeicosatetraenoic acid may be considered a biomarker delineating the pro-inflammatory state of tumor tissues from metastatic CRC [95]. Nevertheless, quite recently, a multicentre, randomized, double-blind, placebo-controlled trial revealed that EPA as well as aspirin did not reduce the risk of colorectal adenoma development [96], pinpointing the inefficacy of ω-3 treatment in this study.

Consistently, in another more recent study on a large Swedish cohort of middle-aged women, the authors showed no overall association between ω-3 fatty acid dietary intake and CRC risk. Nevertheless, they observed that the risk of rectal cancer was reduced in association with high ω-3 DHA dietary intake [97], thus suggesting that ω-3 beneficial effects do not depend on all derivatives of ω-3 PUFAs but that they can be mediated by specific DHA-derived SPM classes.

There is some epidemiological evidence that ω-3 PUFA supplementation rather than dietary ω-3 PUFA intake is associated with improved CRC outcomes. The VITamins and Lifestyle (VITAL) cohort study collected data on ω-3 PUFA supplement use as well as dietary ω-3 PUFA intake demonstrated that fish oil supplement users (≥ 4 days per week for ≥3 years) had 49% decreased CRC risk compared with nonusers [98]. Moreover, other studies have shown not only that fish oil supplement alters markers of inflammatory and nutritional status in CRC patients [99] but also that ω-3 PUFA supplementation has beneficial effects on either efficacy or tolerability of traditional chemotherapy [100].

Different studies tried to correlate shorter-chain PUFAs (i.e., LA and ALA) and longer-chain PUFAs (i.e., AA, EPA, and DPA) with cancer risk. Here, the authors indicated that higher levels of circulating shorter-chain PUFAs were associated with reduced CRC risk whereas longer-chain PUFA levels correlated with an increased CRC risk [101]. Likewise, results regarding ω-3 PUFA inefficacy have been also reported in patients with chronic inflammatory conditions [102]. These discrepancies may be due to the different treatment regimens (prevention or treatment of a fully established disease) used in these studies, to some defects in routes metabolizing ω-3 or ω-6 PUFAs, or to their incapability to reach the site of inflammation [20]. Consistently, a systematic review and meta-analysis of clinical studies indicated that ω-3 PUFAs were beneficial for CRC patients in terms of anti-inflammatory effects but that these results were dependent on the duration, dose, route of PUFA administration, and the presence of concomitant therapies, which may impact the efficacy of ω-3 PUFA administration [103].

Despite these controversial results, single PUFA-derived mediators and their metabolizing route(s) in the orchestration of CRC pathogenesis are still worthy of investigation. This is possible thanks to the use of in vivo and in vitro models of CRC. Here, the control of specific ω-3 PUFA functions and pathways is more feasible than in clinical studies, thanks to homogenous backgrounds and more straightforward experimental designs. 

One of the first studies was performed by Oshima and colleagues more than 20 years ago [104]. By using the APC^Δ716^ mouse model of spontaneous intestinal carcinogenesis, the authors pointed out the importance of pro-inflammatory AA-derived metabolites via COX-2, such as PGE2; indeed, elevated COX2 expression is found in greater than 90% of CRCs [104,105,106], associated with high levels of PGE_2_, which drives pro-tumorigenic proliferation, migration, and invasion and promotes an immunosuppressive tumor microenvironment beneficial for tumor growth. Furthermore, the genetic deletion of COX2 or its inhibition reduced the frequency and size of intestinal polyps [107], indicating that lipid mediators derived from this enzyme might be fundamental for CRC initiation. Some years later, ω-3 EPA and DHA have been shown to block COX-2 expression in colon cancer cells [108], evidence that could explain, at least in part, their beneficial role in CRC.

It is noteworthy that ω-3 PUFAs (EPA and DHA) and ω-6 AA share the same downstream metabolic pathways. The COX-2, LOX, and CYP450 pathways can produce lipid mediators starting either from ω-6 AA or from ω-3 DHA and EPA, usually producing pro-inflammatory or pro-resolving molecules, respectively. Since COX, LOX, and CYP450 metabolize all these substrates with the same affinity, the final and total amount of pro-inflammatory or pro-resolving molecules depends on the level of the starting PUFA precursor (AA, EPA, or DHA) that competes with the others for its metabolism [19,20]. As a consequence, the higher the intake of ω-3 PUFAs, the higher the level of beneficial lipids that are produced. In line with this, DHA not only was found to block the AA-induced proliferation of human colon cancer cells [109] but also was able to induce their apoptosis [110,111], since it displays, like the other ω-3 PUFAs, a high unsaturation degree of the carbon chain, thus being susceptible to free radical attack and peroxidation. 

Another function of ω-3 PUFAs that has been recently highlighted and that deserves attention is represented by their ability to influence epigenetic mechanisms, regulating the expression of genes key for cancer growth; ω-3 PUFAs were indeed found to enhance apoptosis via specific promoter methylation both in vitro and in vivo [112]. 

After the first studies on mice [107], many others have emerged with a growing body of evidence depicting ω-3 as beneficial: for example, the endogenous conversion of ω-6 to ω-3 PUFAs in fat-1 transgenic mice repressed colorectal tumor cell growth and tumor burden in the genetic APC^Min/+^ model of CRC. Accordingly, ω-3 DHA and EPA were found able to inhibit proliferation and to promote apoptosis in models of CRC [113,114]. 

One of the possible mechanisms explaining the effect of ω-3 PUFAs was designed by Han and colleagues. In vitro and in vivo studies revealed that DHA administration induced apoptosis via the downregulation of survivin and Bcl-2 and the upregulation of Bax together with the inhibition of β-catenin complex dissociation in both HCT116 colon carcinoma cell line and the AOM/DSS model of cancer [115]. The proapoptotic function held by the DHA was demonstrated to be autocrine TNF-α-dependent both in vitro and in vivo [110]. 

Similar results were obtained in AOM/DSS-induced tumor-bearing mice treated with EPA, which was found to restore the loss of Notch signaling occurring in this model and was known as the basis of colorectal carcinogenesis [116]. Moreover, EPA was also able to reduce liver metastasis in mice undergone intrasplenic injection of syngeneic MC-26 mouse CRC cells [117].

This collection of studies (Table 1) has opened a promising, new field of research, where specific lipid molecules found their positions as actors of the resolution of cancer-associated inflammation. In the following sections, we will highlight studies pointing out resolving ω-3- and ω-6-derived lipid mediators as possible molecules for cancer treatment.

## 6. Specialized Pro-Resolving Lipid Mediators in Colorectal Cancer

Despite the discrepancies observed in clinical studies, the role of ω-3 DHA and EPA as inhibitors of colorectal carcinogenesis, at least in animals and human cell lines, is now well accepted. Likewise, much evidence has been coming out in the last years regarding the possible role of DHA-, EPA-, and AA-derived SPMs in counteracting the CRC-associated inflammation and in promoting its resolution. Although no clinical studies have pointed out their beneficial effects in humans yet, some data highlighted the importance of these lipid mediators in abating tumor growth and progression in preclinical models of CRC, often pointing out some of them as potential biomarkers of CRC risk and staging.

### 6.1. SPMs Derived from ALA 

**Figure 3:**
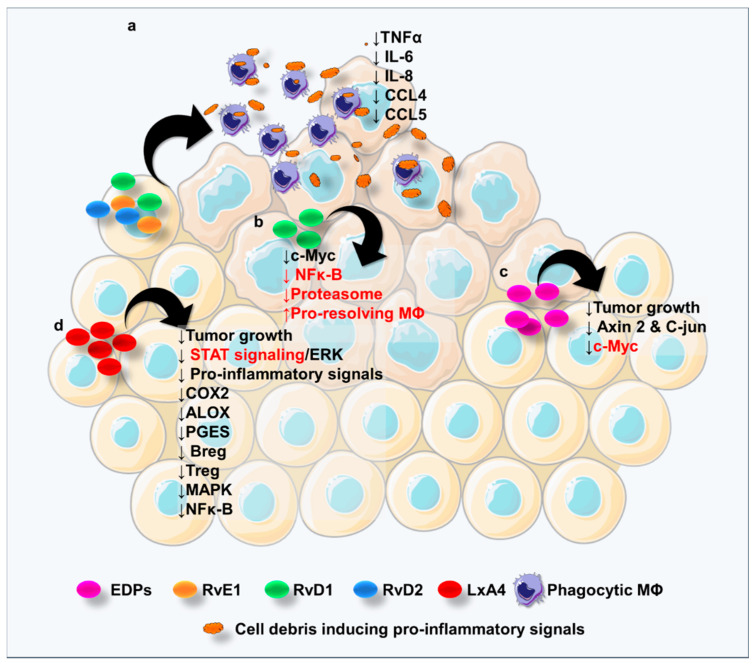
Pro-resolving lipid mediators in CRC. Cartoon showing the CRC-associated pro-resolving lipids. (**a**) RvE1, RvD1, and RvD2 block the cell debris-induced TNFα, IL-6, IL-8, CCL4, and CCL5 release. (**b**) RvD1 inhibits c-Myc expression by tumoral cells through the inhibition of NFκ-B signaling, proteasome, and pro-resolving MΦ. (**c**) CYP450-metabolized epoxydocosapentaenoic acids (EDPs) inhibit tumor growth and the expression of pro-oncogenic c-Myc, Axin2, and C-jun genes. (**d**) LXA4 inhibits tumor cell growth, and its beneficial role is mediated by Breg cell inhibition through inactivation of Extracellular Signal-Regulated Kinases (ERK) and Signal Transducers and Activators of Transcription (STAT) signaling, finally decreasing Treg cell in tumor-bearing mice; LXA4 upregulation is associated with the decreased synthesis of pro-inflammatory mediators and suppressed expression of COX-2, ALOX5, and mPGES. LXA4 reduces tumor growth by inhibiting the Mitogen-Activated Protein Kinase MAPK and NF-κB pathways. The illustration was made with SMART Servier Medical Art free images (https://smart.servier.com/).

Several results come from studies elucidating the role of RvDs in CRC. Tumor-promoting cell debris, including those derived from colon cancer cells, generated after chemotherapy can be cleared by phagocytic macrophages activated by RvE1, RvD1, and RvD2 treatment, thus preventing tumor recurrence. These Rvs were found to block the cell debris-induced TNFα, IL6, IL8, CCL4, and CCL5 release (Figure 3a), pointing out the protective role of these lipid mediators in the context of CRC [118]. Nevertheless, plasma levels of RvD1 as well as lipoxin A4 (LXA4) were not directly correlated with reduced adenoma risk in a trial exploiting aspirin to prevent colon adenomas [119], suggesting that these specific SPMs in the plasma cannot be used as predictors of CRC occurrence. 

The indirect correlation between RvD1 levels and tumor staging in patients with colon cancer has been recently depicted: the higher the stage, the lower RvD1 levels were found [120]. The antitumor activity of RvD1 was observed to act through the inhibition of the G-protein coupled formyl peptide receptor 2 (ALX/FPR2)-mediated c-Myc expression in either TNFα-stimulated normal colon cells (Figure 3b) or colon cancer cell lines via the attenuation of NF-κB signaling and proteasomal degradation and the stimulation of resolution macrophages [121].

Although DPA-derived metabolites, named protectin D1*_n_*_−3 DPA_ (PD1*_n_*_−3 DPA_) and resolvin D5*_n_*_−3 DPA_ (RvD5*_n_*_−3 DPA_), have been found to suppress chronic inflammation [19,122] and their role in cancer, and CRC has not been elucidated yet. So far, only one study reported that RvD5 may be useful for attenuating chemotherapy-induced peripheral neuropathy (CIPN), a rising health concern in cancer survivors [123].

Concerning the epoxides metabolized via CYP450, a study showed MC38 colorectal cancer cell line implants in mice to grow less rapidly when the animals were fed with a ω-3 PUFA-enriched diet; furthermore, the CYP450-metabolized ω-3 derivative epoxydocosapentaenoic acids (EDPs) were found to be responsible for tumor growth suppression in vivo, with the concomitant reduction of pro-oncogenic c-Myc, Axin2, and C-jun genes in tumor tissues [124] (Figure 3c). 

Although maresins have been found beneficial in chronic inflammation [125], no evidence is available for them to be effective in cancer and consequently in CRC. Maresins displayed the ability to limit polymorphonucleate infiltration [126], to enhance macrophage-driven clearance of the damaged tissue [127], to suppress oxidative stress [128] and to reduce pro-inflammatory mediators [129]. With all these inflammation-specific processes hallmarks also in cancer, further studies endowing maresins with anti-CRC functions will be warranted. 

### 6.2. SPMs Derived from LA

In a subcutaneous injection of a colorectal cancer cell line in mice, the LXA4 agonist BML-11 was shown to inhibit tumor cell growth, demonstrating the antitumor activity of LXA4 in vivo. Moreover, the authors showed that the beneficial LXA4 role was mediated by Breg cell inhibition through inactivation of Extracellular Signal-Regulated Kinases (ERK) and Signal Transducers and Activators of Transcription (STAT) signaling, finally decreasing Treg cell in tumor-bearing mice [130] (Figure 3d).

Of note, PUFA treatment of colorectal LoVo and RKO cancer cells in vitro increased LXA4 levels, with a concomitant decreased synthesis of pro-inflammatory mediators and suppressed expression of COX-2, ALOX5, and mPGES [131] (Figure 3d).

In line with this study, recently, Liu and colleagues showed that serum LXA4 levels were lower in CRC patients by comparison with control, accompanied by the increase of serum levels of IL1B, IL6, CXCL8, TNFα, and CCL2. The authors investigated also the role of LXA4 in vivo upon subcutaneous injection of the CT26 colorectal cancer cell line in mice and observed that this mediator was able to slow tumor growth by comparison with control animals by inhibiting the Mitogen-Activated Protein Kinase (MAPK) and NF-κB pathways [132] (Figure 3d).

Altogether, this evidence depicts SPMs as in charge, at least in part, of resolving CRC inflammation. However, more efforts are needed to finely dissect the efficacy and the intracellular mechanisms through which SPMs counteract the CRC-associated inflammation. In the next section, we will point out some discoveries elucidating lipid-activated receptors, which ultimately might lead to uncovering novel therapeutic targets or innovative agonists promoting the physiological process of the resolution of tumor-associated inflammation.

## 7. PUFA Receptor-Mediated Signaling in CRC

PUFAs bind to and mediate their functional effects through specific membrane-bound receptors, the G-protein coupled receptor 40 (GPR40) and 120 (GPR120) [133]. Activation of these receptors has many health benefits, including the modulation of inflammatory responses and energy intake [134]. G-protein activation by GPRs triggers the production of hundreds or even thousands of second messenger molecules. Common targets of activated G proteins are adenylyl cyclase, which catalyzes the synthesis of cyclic Adenosine Monophosphate (cAMP) from ATP molecules [135], and phospholipase C, a membrane-associated enzyme catalyzing the synthesis of diacylglycerol (DAG) and inositol trisphosphate (IP3) from the membrane lipid phosphatidylinositol. This pathway is crucial for a variety of human biological processes [135]. 

GPRs are expressed by various cell types at different stages of differentiation and contribute to tissue repair, inflammation, angiogenesis, as well as normal and tumor cell growth. In fact, in many cells, mitogen elements (such as thrombin, lysophosphatidic acid, gastrin-releasing peptide, and prostaglandins) stimulate cell proliferation by binding and activating their G protein-coupled receptors (GPCRs) [136].

Since GPR40 and GPR120 are sensors of and modulate responses to a variety of ω-3 and ω-6 PUFA-derived lipid mediators, these receptors likely mediate divergent outcomes in human cancers, as shown in CRC [137]. Unfortunately, to date, few studies examined the roles of GPR40 and GPR120 in CRC cells. While only a single study reported an involvement of GPR40 in CRC development, showing that activation of this receptor may be associated with the progression and prognosis of CRC [138], more pieces of evidence pointed out a contribution for GPR120 in colorectal carcinogenesis, even if conflicting. As an example, Wu and colleagues found that the expression of *FFAR4*, the gene encoding for GPR120, is upregulated in human CRC tissues compared to adjacent noncancerous areas [139] and that the expression of the receptor increases as the clinical stage of cancer advanced, with 100% of stage III histological grade CRCs expressing high levels of GPR120. The same authors found GPR120 expression significantly increased in eight human CRC cell lines in comparison with normal colon cell lines and its expression correlated with tumor progression. Besides, activation of GPR120 signaling in human CRC promoted angiogenesis and tumor growth in vitro and in vivo, along with the enhancement of CRC cell motility and induction of epithelial-to-mesenchymal transition [139]. Results from Hopkins et al., using the Caco2 colorectal cell line, also indicated that GPR120 agonists activate Akt in these cells and do not inhibit growth factor response [140] in contrast to their inhibitory effects in prostate and breast cancer cells [140,141].

On the contrary, Zhang and colleagues demonstrated that GPR120 binding to DHA and EPA suppresses in vitro cell proliferation and promotes apoptosis in CRC cell lines treated with ω-3 PUFAs, through the GPR120-mediated activation of the canonical hippo pathway [142]. During AOM/DSS-induced colorectal carcinogenesis, animals fed with a ω-3-enriched diet showed tumors reduced in size, number, and activation of the Hippo pathway compared to control diet-fed mice [142], suggesting that GPR120 may mediate the antitumoral effect exerted by ω-3 PUFAs in CRC.

The two studies from Wu and Zhang showing pro- and antitumor effects, respectively, clearly delineate a role for GPR120 in CRC and offer contrasting views on the involvement of the receptor in this disease. Notably, while the work of Wu and colleagues [139] did not detect the expression of GPR40 in either human CRC tissues or cell lines, the results from Zhang did [142]. These misleading results might be due to the use in these studies of nonselective FFA1/FFA4 agonists such as EPA, DHA, and GW9508. Thus, future investigations exploiting selective agonists should better delineate the role of GPR120 in CRC. 

Interestingly, recent findings showed that EPA, DHA, and AA elicit the same signaling events but with different kinetics and efficiency, through GPR120 in Caco-2 CRC cells. Both ω-3 and ω-6 PUFAs were found to activate independent intracellular signaling events in a colorectal cancer cell line via GPR120. Thus, EPA, DHA, and AA were able to activate these pathways with different kinetics and intensity, promoting the idea that, within the tumor microenvironment, ω-3 or ω-6 PUFAs may exert anti- or protumoral effects based on their concentration [143]. 

Altogether, these findings suggest that GPR40 and GPR120 might partially explain some of the anticancer effects elicited by ω-3 PUFAs. However, the varying responses to GPR120 activation in different cancer cell types, tissues, and models demonstrate that further studies exploring the role of this receptor in CRC are needed.

## 8. Pharmacology and Diet as a Possible Approach to Enhance the Lipid-Mediated CRC-Associated Resolution of Inflammation

The field of lipid-mediated resolution of inflammation in cancer is growingly attracting interest, mostly because it opens new possibilities to develop drugs able to target and activate the pro-resolving pathways, without subverting the inflammatory processes naturally occurring in the body and necessary to counteract external insults and to heal tissues. The most successful way could be the administration of SPMs to locally resolve CRC-associated inflammation. However, due to the quick metabolic inactivation, SPMs undergo rapid degradation and become biologically ineffective [144]. To overcome this issue, some more stable analogs have been synthesized. As an example, 15R/S-methyl-LXA4, 16-phenoxy-LXA4, and 15-epi-16-phenoxy-LXA4 were found to potently inhibit neutrophil transmigration across intestinal epithelial cells and adhesion to endothelial cells [144]. However, the employment of the SPMs in terms of dosage, duration of treatment, and route of the administration remains to be elucidated. Interestingly, some drugs currently in use, besides the ability to block the production of pro-inflammatory prostaglandins, were shown to specifically activate pro-resolving molecules and to have a role in the resolution of cancer-associated inflammation. For example, a series of LXs and RVs are formed at a low dosage of aspirin, which can switch COX-2 activity from producing PGE2 to pro-resolving aspirin-triggered LXs and RVs. These aspirin-triggered SPMs were demonstrated to resolve the cancer-promoting inflammation in mice, finally reducing tumor growth and metastasis, through the clearance of tumor cell debris [145]. Aspirin was also shown to promote the formation of antiproliferative 15-epi-LXs by epithelial cell-leukocyte interactions [146]. These studies suggest how aspirin, which is indeed effective in CRC treatment and prophylaxis, may exert its beneficial role also activating specific pathways of the resolution of CRC-associated inflammation. 

Diet may be another way to treat CRC and to dampen the CRC-associated inflammation. As we discussed above, the PUFA-enriched diet may ameliorate CRC relapse or may reduce CRC risk, even though discrepant results may lead to non-consistent conclusions, at least in clinical studies. 

Dietary habits determine the high or low rate of intake of nutrients that can be beneficial in CRC management. Among these nutrients, calcium was proposed as involved in the modulation of CRC-related cell signaling pathways [147]. Compelling evidence suggests an anticancer activity exerted by vitamin D, endowed with anti-inflammatory, immune regulatory effects, and antitumor activity [147,148]. In mice with colitis, consumption of a high vitamin D diet attenuated inflammation, suggesting that vitamin D may have an important role in colitis-associated carcinogenesis [149] and may prevent also neoplastic lesions [150]. It is yet to be elucidated whether the antitumor activity held by vitamin D prompts the production of pro-resolving molecules. 

Finally, among the most convincing pieces of evidence regarding the nutrients as pro-resolving molecules sources, fibers are likely to be the best candidate, since the bacterial fermentation of fibers in the intestine leads to the production of the short-chain fatty acids, with anticancer, immune-modulatory, and anti-inflammatory effects [147]. 

Conclusively, because of this promising evidence, the control of the inflammation through specific drugs or nutrients has a strong rationale, and dietary-based strategies for CRC prevention and management are worthy of further investigation. However, some points remain to be elucidated: (*i*) how nutritional factors influence the risk of cancers with distinct etiologies; (*ii*) which treatment regimen should be used to interrupt or reverse carcinogenesis by promoting the resolution of inflammation; and (*iii*) whether dietary components directly stimulate the production of SPMs and/or interact with other host factors to influence CRC pathogenesis. Although the mechanisms are unknown yet, this field exploiting diet to resolve CRC-associated inflammation has plenty of opportunities to develop novel therapies alone or in combination with the first line protocols for the treatment of CRC patients. 

## 9. Conclusions

The intrinsic connection between inflammation and CRC led to the use of many anti-inflammatory drugs, such as NSAIDs, that have become important for the prevention and treatment of CRC by inhibiting COX enzymes and, consequently, AA synthesis [11,13]. Despite their anti-inflammatory properties, NSAIDs display many side effects, including gastrointestinal ulcerations and kidney dysfunction [151]. Thus, the development of alternative therapies may be of help. This is one of the reasons why SPMs have been recently proposed as possible anticancer molecules, based on the rationale that the enhancement of pro-resolving pathways, without subverting naturally occurring pro-inflammatory processes, may facilitate compliance in patients with cancer, including CRC [17]. 

As we reviewed above, a ω-3 PUFA-enriched diet displayed efficacy in experimental models of CRC, although misleading results came from clinical studies, where these PUFAs sometimes did not show beneficial effects. This might be partly due to the complex metabolic routes which these precursors undergo. As discussed earlier, AA, DHA, and EPA compete for the same enzymatic routes, leading to pro-inflammatory (AA-derived, such as prostaglandins) or pro-resolving (DHA- and EPA-derived) mediators, depending on the initial precursor concentrations [19,20]. Also, they can activate the same receptors (i.e., GPR120) with different downstream signaling, further corroborating the idea that ω-3 or ω-6 PUFAs may exert anti- or protumoral effects based on their concentration [137]. 

Additionally, the cellular uptake and metabolism of these PUFAs may be dysfunctional in pathogenic conditions. For example, endothelial DHA absorption and metabolism was found impaired in colons from patients with ulcerative colitis (UC). Thus, the UC gut vasculature was not able to properly produce DHA-derived epoxides [20], leading to a failure in the resolution of chronic intestinal inflammation.

Hence, it is reasonable to argue that also in CRC the inefficacy of the treatment with SPM precursors (ω-3 PUFAs) may generate contrasting results because of dysfunctional ω-3 PUFA metabolic routes carried by CRC patients and those not identified yet. Another important concern is the treatment regimen in clinical studies (prevention or treatment of a fully established disease) that may alter the outcome [20]. Last but not least, exogenously administered ω-3 PUFAs and its derivatives might not reach the site of action compared with the endogenously generated compounds in some cohorts of CRC patients, ultimately resulting ineffective. 

Resolvins, protectins, maresins, and lipoxins may be of importance in CRC treatment because of their ability to ameliorate intestinal carcinogenesis both in vitro and in vivo. To date, although no clinical data are available for demonstrating the efficacy of SPMs in CRC patients, experimental observations render SPMs a promising field for further studies in humans. In this regard, it would be of importance to identify the SPM-specific dosage, route of administration, and timing of treatment as well as drugs or nutrients that can foster the resolution of CRC-associated inflammation. This could help to set up a robust clinical study and to straightforwardly establish an effective line of intervention for CRC patients, eventually avoiding anti-inflammatory treatment side effects and promoting the SPM-mediated resolution of the CRC-associated inflammation. 

## Figures and Tables

**Table 1:** A table listing the clinical and preclinical studies investigating the role of ω-3 polyunsaturated fatty acids (PUFAs) in CRC.

Study Reference	Type of Study	Outcome
Sasazuki et al, 2011; Norat et al, 2005; Hall et al, 2008; Aglago et al., 2019 [55,56,57,58]	Clinical prospective studies	ω-3/CRC risk inverse correlation
Tutino et al., 2019 [58]	Clinical observational study	High ratio of ω-6/ω-3 as metastatic CRC biomarker
Hull et al., 2018 [59]	Clinical, multicentre, randomized, double-blind, placebo-controlled, 2 × 2 factorial trial	EPA does not reduce CRC risk
Shin et al., 2020 [60]	Clinical prospective study	DHA intake reduces CRC risk
Khankari et al., 2020 [61]	Clinical observational study	Shorter-chain PUFAs→reduced CRC risk; longer-chain PUFA levels→increased CRC risk
Oshima et al., 1996 [64]	Preclinical study on APC^Δ716^ mouse model	COX-2 derived AA lipids are essential for tumor growth
Calviello et al., 2004 [65]	In vitro study on colon cancer cells	EPA and DHA block COX-2 expression
Fluckiger et al., 2016; Siddiqui et al., 2008 [67,68]	In vitro study on colon cancer cells	DHA blocks AA-induced proliferation and apoptosis
Sarabi et al., 2019 [69]	Preclinical study on APC^min/+^ mouse model and in vitro study on colon cancer cells	ω-3 PUFAs enhance apoptosis via specific promoter methylation
Liu et al., 2016 [70]	Preclinical study on APC^min/+^ mouse model	ω-3 PUFAs repressed tumor growth and burden
Barone et al., 2014 [71]	Preclinical study on APC^min/+^ mouse model	ω-3 PUFAs repressed tumor growth via apoptosis inhibition
Han et al., 2016 [72]	In vitro study on colon cancer cells and preclinical study on AOM/DSS-treated mice	DHA induces apoptosis and reduces tumor growth
Piazzi et al., 2014 [73]	Preclinical study on AOM/DSS-treated mice	EPA reduces tumor growth by restoring Notch signaling
Hawcroft et al., 2012 [74]	Preclinical study by intrasplenic injection of mouse colon cancer cells	EPA reduces liver metastasis
